# Delivering child health interventions through the private sector in low and middle income countries: challenges, opportunities, and potential next steps

**DOI:** 10.1136/bmj.k2950

**Published:** 2018-07-30

**Authors:** Phyllis Awor,, Stefan Peterson,, Meenakshi Gautham,

**Affiliations:** 1 Makerere University School of Public Health, Kampala, Uganda; 2 London School of Hygiene and Tropical Medicine, London, UK; 3 Uppsala University, Sweden; 4 Unicef, New York, USA; Correspondence to: P Awor pawor@musph.ac.ug

## Abstract

Universal health coverage requires both the public and private sectors to ensure quality, equity, and efficiency in health systems, say **Phyllis Awor and colleagues**

Private health providers are an important source of treatment for common childhood illnesses in low and middle income countries. A recent analysis of 70 countries showed that the private sector provides 63% of treatment for fever or cough and 67% of treatment for diarrhoea for sick children.^1^ Care seeking for key maternal health services, including institutional delivery (38%), antenatal care (30%) and modern contraceptives (39%), is lower^1^ but still significant. In this case the private sector is responsible for providing one third or more of health services that can affect mother and child survival. In addition, most community level management of childhood illnesses in low income countries is through small (one or two rooms) private clinics and the retail health sector (drug shops and pharmacies).^2^ ^3^ This high use of private health providers for management of common childhood illnesses raises questions about the conventional emphasis of critical child healthcare in the public sector.

Health systems need to maximise health outcomes and deliver equitable, inexpensive, good quality services to entire populations. In considering universal coverage, the performance of the private healthcare sector is often assessed in three ways: quality of care, equity, and efficiency. This information is used to compare public and private sector contributions and progress towards universal health coverage. Various systematic reviews highlight poor quality of care in both the public and private sector in low and middle income countries, underlining the need for improvement throughout the health system.^2^ ^4^ Meanwhile, fair availability of healthcare may be seen as low with private healthcare provision, especially given that most private services are funded directly by patients themselves, favouring the better off.^2^ However, the availability of private providers, which increases the opportunity for the whole population to access care—for example, when public provision is weak—can improve overall fairness of the health system.^5^

In view of the low public financing of healthcare, high out-of-pocket expenditure and the high use of private providers in low and middle income countries, we will be unable to achieve universal health coverage without working with both public and private sectors to ensure quality, equity, and efficiency.^5^

We discuss the opportunities and challenges of working with the private sector for delivery of child health interventions, and propose possible next steps. We draw on the findings of the 2016 strategic review of Integrated Management of Childhood Illnesses (IMCI), coordinated by the World Health Organization and Unicef^6^ and in particular, a systematic literature of review of evidence on working with the private sector, interviews with key informants, and country assessments.

## Challenges of working with the private sector towards public health goals

Working with the private sector for public health goals presents challenges owing to its heterogeneous, multi-layered, and highly segmented structure (table 1). The private health sector can be divided into for-profit and not-for-profit, and further subdivided into formal or informal providers. These providers are widely diverse, ranging from private doctors and nurses to medical and nursing assistants, and even include traditional healers and drug peddlers (table 1). It may be easier to form a partnership with large private facilities and hospitals (both for-profit and not-for-profit) to improve access and quality of care. However, many patients in low and middle income countries also seek care from the informal and retail sector, where regulation is weak.^2^ Frequent recourse to the informal private sector and weak regulation make it complicated for governments to make firm decisions about whether to prohibit, constrain, or encourage the sector.^7^

**Table 1:** Heterogeneous and multilayered composition of the private sector

	Formal	Informal
For profit	▪ Private hospitals/clinics (outpatient care, inpatient care, multispecialty, superspecialty) ▪ Private doctors (general physicians) ▪ Private registered/licensed pharmacies, drug shops and proprietary patent medicine vendors (Nigeria) ▪ Private mobile clinics ▪ Private nurse/paramedic/other formally trained health worker ▪ Public-private mixed—eg, village doctors and village clinics in China, and public doctors working privately in India ▪ Publicly owned hospitals and public providers with high user fees ▪ Private non-biomedical providers and facilities—eg, formally qualified AYUSH practitioners in India and practitioners of Chinese medicine/integrated medicine in China	▪ Unregistered pharmacies and drug shops ▪ Public sector frontline health workers providing private healthcare beyond their scope of work, for a fee ▪ Private practitioners of allopathic medicine; may be commonly referred to as small doctors or private doctors in India and village doctors in Bangladesh. ▪ Traditional healers ▪ Friends and relatives ▪ Drug peddlers and vendors
**Not for profit**	▪ Non-governmental hospitals/clinics—eg, LV Prasad Eye Hospital, India ▪ Faith based hospitals such as mission hospitals ▪ Community based depot holders and other fieldworkers ▪ Public-private partnerships between governments and NGOs to deliver health services such as mobile clinics or delivery centres in hard to reach areas	▪ It is possible for not-for-profit entities to function informally—eg, small charities and unrecognised spiritual healers

AYUSH=Ayurveda, yoga and naturopathy, Unani, Siddha, and homeopathy; NGOs=non-governmental organisations.

Furthermore, failures occur in private healthcare markets—for example, the markets may be unable to adequately provide public goods, and there may be problems with obtaining information from them. The market failures in the provision of public goods relate to inefficient allocation and inadequate supply of goods and services, and charging for services that are supposed to be free—for example, immunisation. This especially affects the poor, who may not be able to afford or access private services. Imperfect information and information asymmetry (providers know more than the patients) also exist in health markets. This can generate adverse selection in insurance and provider induced demand for services,^8^ which coupled with profit incentives may be stronger in the private sector.

Service quality (which includes drug availability and patient satisfaction) is often reported to be better in the private than the public sector. Private providers may be more responsive as they have greater motivation to encourage patients to return and fewer financial restrictions than public providers.^4^ However, technical quality (including provider competence and adherence to treatment guidelines) may be lower in the private sector.^4^ ^5^

These characteristics of the private sector require government to make a careful analysis of health system bottlenecks and a plausible assessment of how a public-private partnership will improve health outcomes. Governments must ensure equity and access to care; affordability; quality of care within the diverse private sector; and adequate regulation. However, gross public sector inadequacies exist in low and middle income countries. These include lack of medicines, inaccessible health facilities, health worker shortages, and high out-of-pocket expenditure. All these factors justify the search for ways to improve service delivery in general, and also a careful consideration of public-private partnerships. These may be more appropriate in some settings (eg, informal urban settlements) than others, and must be assessed so that they do not drain public sector funds and clients, where a dominant public sector provision is desired.

## Opportunities of working with private health providers

The important role of the private sector in the future of child health delivery was well articulated in global key informant interviews and country assessments.^6^ About half of the key informants stated that cooperation with the private sector is essential for improving child health and cannot be overlooked because these providers are often much closer to the community.^6^ This was particularly emphasised in the assessments for Nigeria and India, where the private sector is much used. Until now, the private sector has been largely neglected owing to institutional barriers that have prevented its inclusion, the weak ability of the public sector to perform an effective stewardship role, and a general mistrust of the sector.

Over the past 10 years, the implementation of programmes which include the private sector has steadily increased.^9^ Different approaches have been used when working with private health providers in order to improve quality of care, increase availability of goods and services, and to ensure affordability, equity and coverage of health services. These strategies include regulation, accreditation, contracting out, social marketing, social franchising, use of vouchers, and pre-packaging of drugs.^7^ ^10^ Box 1 presents existing strategies for working with private health providers and summarises the evidence for each strategy. For child health, contracting out (particularly in fragile states), use of vouchers, accreditation, social marketing and social franchising are the commonly used approaches (box 1).

Box 1 **Approaches for working with private health providers for the delivery of child health interventions**

**Social marketing**

Social marketing is the application of commercial marketing to social and health problems, in order to increase population coverage of effective and affordable interventions.^11^ It may include mass promotional activities, branding, labelling, pre-packaging and subsidy of public health products. It has been used to create demand for health products, including contraceptives, mosquito nets^10^ and malaria medicines, even through for-profit channels, where these commodities are often subsidised. It has also been used to positively influence health related behaviour, including immunisation, use of oral rehydration therapy and HIV prevention.

Reviews on commodity social marketing highlight the importance of an integrated package, including mass media, training of healthcare providers, patient outreach and the concurrent supply of the commodities and services being promoted, for the strategy to be effective.^12^

**Social franchising**

A franchise is a contractual arrangement between a health service provider and a franchise organisation, which aims to improve access to quality and price controlled services. Franchisees are trained in standardised practices for which prices are predefined, and they benefit from advertising the logo or franchise name. In return, franchisees may be required to comply with a minimum sales volume, quality standards, and pay a membership fee to the franchiser. The franchise organisation (a government or donor-sponsored non-governmental organisation) monitors providers and subsidises the network.

Franchising is associated with increased numbers of clients, patient satisfaction, physical accessibility, and improved quality. Findings related to healthcare use—namely, the effect on health, efficiency, and provider outcomes—are mixed. Further research is needed to elucidate the effect of franchising for quality, health impact, equity, cost effectiveness, and the value of franchising in other healthcare sectors like child health.^13^ ^14^ ^15^

Numerous social franchising programmes already exist around the world, providing an opportunity to expand access to care rapidly and standardise and improve the quality of care. This could form the basis for evaluation of private sector initiatives, provided that evaluation is built into further expansion of the social franchises.

**Vouchers**

Vouchers are a form of demand-side subsidy that recipients use as part or full payment for a product or service from identified providers. Distribution of vouchers can be targeted—for example, to the poorest households or pregnant women. Vouchers could be competitively redeemed through use of different providers, or non-competitively assigned to a particular provider.^11^ The use of vouchers has been shown to improve access to maternal healthcare, although the problem of misuse of the subsidy has also been identified.^16^

**Accreditation**

Accreditation is a strategy to improve and control quality of services provided at facility level through oversight by an independent quality control evaluation body (government or a non-governmental organisation). It may include training providers in standardised practices.^10^ Accreditation is similar to franchising, although it is often voluntary, unlike the contractual relationship between franchisee/franchiser. Accreditation has been successfully used for WHO laboratory quality control, and within the pharmaceutical sector, to improve the quality of drug dispensing.

**Contracting out**

Contracting out is a purchasing mechanism used to acquire specified services, of defined quality, at an agreed price, from a specific private provider and for a specific period of time. Governments may purchase clinical or non-clinical services from private providers to complement public provision. Contracting out has been shown to be effective at increasing access and use of health services, particularly in conflict or fragile states.^17^ ^18^

**Pre-packaging**

Drugs may be packaged in predefined doses adequate for the targeted population group and length of treatment regimen.^10^ This is particularly useful for paediatric medicines and is used to improve provider and patient adherence to treatment regimens. Pre-packaging is sometimes used with commodity social marketing.

**Training**

Training activities are often integrated into other strategies, including franchising, accreditation, and social marketing interventions. They can take various forms, including formal training sessions, vendor-to-vendor education, distribution of guidelines and job aids.

Literature reviews consistently show that provider training is insufficient to change practice or improve quality of care in the private sector.^19^ ^20^ This is because various other factors, including supply chain management, patient expectation, profit motivation, etc, affect provider practice more strongly. Hence approaches that combine provider training with consumer education yield better results.

**Regulation**

Regulatory interventions are used to set up and ensure adequate technical quality of service providers. Regulation involves setting rules, sanctions, and ensuring adequate enforcement. Basic regulatory frameworks exist in most countries, particularly for pre-service training, registration and licensing requirements for health workers and premises. Pharmaceutical market regulation aims to limit the availability of harmful drugs and unregistered products, minimise drug misuse, control the sale of specific drugs through prescriptions and regulate drug manufacture and importation. Regulation has a crucial balancing role within the private sector, although, inadequate resources are typically allocated for monitoring and enforcing regulations. Co-regulation with professional associations, civil society, and communities can provide additional benefit.

Social marketing involves applying principles of commercial marketing to social health problems, whereas a social franchise is a contractual arrangement between a health service provider and a franchise organisation, which aims to improve access, quality of healthcare, and price regulation^11^ (box 1). The intermediary for these strategies is often a non-governmental organisation with donor funding, raising questions about sustainability. Alternatively, management of these strategies may be through ministries of health and education. The promotional and accreditation aspects are worked out, but it is generally harder to ensure that the quality of care delivered meets required standards.

The employment of social franchising is growing. In 2013 alone, Viswanathan et al reported the existence of social franchises in over 40 countries in Africa, Asia, and Latin America, with over 95 000 providers operating as part of social franchised networks.^21^ These franchises are often led by international organisations such as Population Services International, Marie Stopes International and FHI 360. They take various preventive and lifesaving services related to family planning, maternal and child health, tuberculosis, and HIV testing to millions of people around the world. Additionally, Integrated Community Case Management (iCCM) is employed for malaria, pneumonia and diarrhoea child survival strategy (box 2), through certain franchised outlets like those of Population Services International.^24^ The idea is that using private sector incentive mechanisms and supply chains will enable these services to reach communities more effectively than public services. However, formal comparative studies of care provided by franchised networks versus the public sector are generally lacking.

Box 2 **The need for Integrated Management of Childhood Illnesses (IMCI) within the private sector**

**IMCI**

Since 1995, IMCI has been the key strategy for treating sick children and improving child survival in countries with high child mortality. IMCI provides guidance on treatment and care for the major childhood illness, including malaria, pneumonia and diarrhoea, and malnutrition. It has three components: improving health worker skills, strengthening health systems, and family and community practices. IMCI has been shown to improve health worker performance and quality of care, but it did not achieve the expected effect on mortality mainly owing to delayed care seeking.^22^

**Integrated Community Case Management (iCCM)**

To improve the treatment seeking practices for sick children under IMCI, community case management was recommended, to complement the health facility based services. Community case management includes treatment of sick children at the community level and promotes timely care seeking and referral to health facilities. iCCM is supported by WHO and Unicef to increase access to care for malaria, pneumonia, and diarrhoea in children aged <5 years.

**Low use of the private sector for integrated management of malaria, pneumonia, and diarrhoea**

In 2014, Awor et al reviewed the literature on experience with iCCM within both the public and private sectors.^23^ They aimed at understanding the degree to which the private sector was used for IMCI. Evaluation studies investigating the effect of introducing an intervention with drugs or diagnostics, for malaria, pneumonia, or diarrhoea, within both the public and the private sector were included. This review found four times as many evaluation studies referring to malaria, pneumonia, or diarrhoea in the public sector (49 studies) as in similar studies within the private sector (13 studies). Most public sector iCCM studies evaluated the introduction of drugs and/or diagnostics for two or more illnesses (malaria, pneumonia, and diarrhoea), while almost all studies in the private sector examined interventions for one disease only, malaria.^23^ The studies were all made within retail drug shops.

These results indicate that the private sector has focused more on interventions for a single disease (especially malaria) and not integrated care. Clearly, the private sector has not been effectively used for integrated child care. This follows the historical pattern of single disease focus in the public sector (starting with home management of malaria), which has now evolved into the iCCM strategy.

To improve rational drug use and quality of care for sick children, the logical next step should be private sector engagement at community level, for integrated service delivery for acute febrile illness in children. This might include provision of alternative appropriate care if the malaria diagnostic test is negative. In this regard, iCCM is an appropriate strategy, which should be further explored.

Two other franchised networks, Living Goods (based in the USA)^25^ and the *Bangladesh Rural Advancement Committee (*BRAC)^26^ are using the iCCM strategy within the private sector in Uganda and Kenya. Working through a network of mainly female community health promoters, the franchised networks use a non-profit entrepreneurial delivery model where the community health promoters earn a margin on product sales and performance based incentives. An evaluation of the Living Goods entrepreneurial model of community health delivery in Uganda found that the intervention reduced the under-5 mortality rate by 25% in comparison with controls.^27^

These examples highlight the opportunity for using existing community based private provider networks to increase access to healthcare and expand the reach and coverage of the IMCI strategy. The quality of care for children in the private sector may also be improved using IMCI and iCCM strategies.

The evidence for social franchising is limited. A 2009 systematic review found no studies meeting the rigorous Cochrane inclusion criteria.^13^ More research is needed to further evaluate the effect of franchising on quality, health, equity, and cost effectiveness and the value of franchising in other healthcare sectors.^13^ ^14^ ^15^ In a 2014 systematic review on IMCI in Africa, Awor and colleagues found that private sector involvement tended to focus on single disease interventions (especially malaria) rather than integrated management of children.^23^ The review found only one study on the IMCI in the private sector,^28^ highlighting the need for better evidence for the ability of the different private sector segments to provide integrated care for children (box 2). To support a programme of child health interventions, evidence needs to be collected through operational research in conjunction with the existing wide scale private provider networks. Donors who support private sector initiatives should also see investment in rigorous research as a priority for gradually increasing the evidence to scale up successful models.

**Fig 1:**
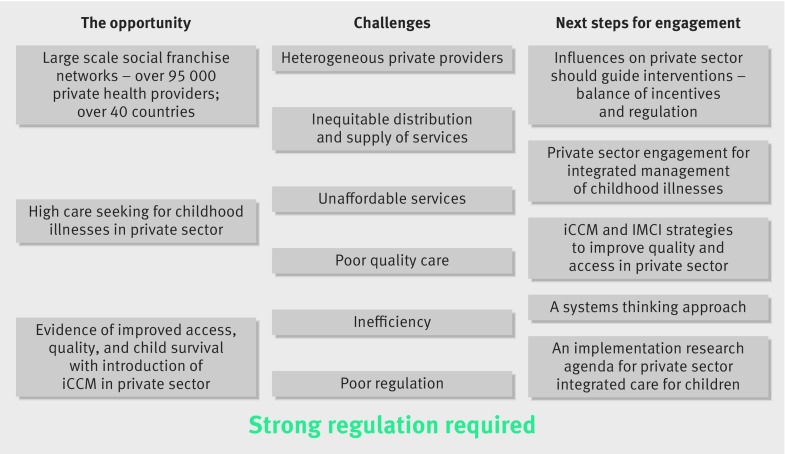
Challenges, opportunities, and next steps for working with the private sector to improve child health outcomes in low and middle income settings. iCCM=Integrated Community Case Management; IMCI=Integrated Management of Childhood Illnesses

Figure 1 provides an overview of the challenges, opportunities, and possible next steps for working with private health providers to improve child health outcomes in low and middle income countries.

## Next steps

Evidence is growing in favour of private sector approaches that are relevant for improving child health outcomes. These include contracting out (for example, of primary healthcare in the post-conflict states of Cambodia and Rwanda),^17^ use of vouchers to increase access to delivery services for poor populations,^16^ accreditation of health facilities and laboratories, social marketing and social franchising. However, for maximum benefit, these strategies must be used in various combinations, as single interventions are generally less effective^19^ in both public and private health sectors. In addition, it is important to support wide scale use of social health insurance to reduce out-of-pocket health expenditure, through pooling contributions from individual community members, governments, and external funding that is available for health in low and middle income countries.

Given the high healthcare seeking in the private sector, governments (which are responsible for the health of their citizens) cannot afford to leave the sector unregulated. The market influences on the private sector should guide the conceptualisation, design and implementation of interventions. There are multiple institutions that influence health markets, many people involved, and an interplay of formal and informal rules within these market systems. For this reason, interventions that focus too narrowly on specific aspects—for example, training—are likely to fail 19 because they do not adequately anticipate and account for complex interactions among the existing stakeholders.^29^ Additionally, health markets exist within the broader health system, which require a “systems thinking” approach to working with private providers.^30^ Systems thinking considers the effects of a particular intervention on other health system building blocks and enables holistic understanding of interactions with the rest of the health system. This increases understanding of both the intended and unintended consequences of private sector interventions.^29^ ^31^

To improve access to healthcare, the next logical step is to include private health providers in community health systems. Private health providers at community level could be used to expand access to integrated care for children with common illnesses, such as malaria, pneumonia, and diarrhoea. The private sector supply chain and incentive mechanisms can be used to ensure availability of drugs and commodities in the community, probably more effectively than through public channels. This is dependent on the type of private sector that exists in a specific setting.

It is possible for IMCI and iCCM strategies to improve quality of care in the private sector, provided that they are adapted for use in the sector. Adaptation should include recommendations for the price of drugs, diagnostics, and any price subsidies; determining who will supervise the private providers in the community; and education of communities about management of childhood illnesses and what to expect in both the public and private sectors. At the same time routine IMCI/iCCM activities and interventions should continue. Conversely, the private sector can improve the reach and coverage of IMCI, given the wide scale of healthcare seeking in the sector. However, there is need for more evidence on the effect of using the iCCM strategy within the private sector on child health outcomes; and how it can be used within existing private sector approaches like social franchising, in conjunction with programmes in different settings. Thus, research into private sector integrated care of febrile illness in children needs to be carried out, in conjunction with private sector programmes, in multiple settings.

**Key messages**

- Care for most children is sought from private health providers in the for-profit sector in low and middle income countries
- Challenges of private sector engagement include its heterogeneous composition, poor quality of care, and inherent market failures
- Challenges have been overcome using existing approaches, such as accreditation, contracting out, use of vouchers, social franchising, and social marketing as well as innovative (although small scale) introduction of the Integrated Community Case Management (iCCM) and Integrated Management of Childhood Illnesses (IMCI) strategies in the private sector, to improve quality, access, and affordability of care
- Inclusion of private health providers in community health systems and an implementation research agenda for private sector IMCI is recommended

## Notes

See www.bmj.com/child-health for other articles in the series.

This article is part of a series based on findings from a global review of Integrated Management of Childhood Illness funded by the Bill and Melinda Gates Foundation. Open access fees were funded by the Health Systems Research Unit, South African Medical Research Council. *The BMJ* peer reviewed, edited, and made the decision to publish the article with no involvement from the Medical Research Council.
